# Elevated NT-proBNP levels are associated with CTP ischemic volume and 90-day functional outcomes in acute ischemic stroke: a retrospective cohort study

**DOI:** 10.1186/s12872-022-02861-w

**Published:** 2022-09-30

**Authors:** Xiaozhu Shen, Juan Liao, Yi Jiang, Yiwen Xu, Mengqian Liu, Xianxian Zhang, Nan Dong, Liqiang Yu, Qingmei Chen, Qi Fang

**Affiliations:** 1 Department of Neurology, The First Affiliated Hospital of Soochow University, Suzhou, 215000 China; 2 Department of Geriatrics, Lianyungang Second People’s Hospital, Lianyungang, China; 3 Department of Neurology, Yancheng Third People’s Hospital, Yancheng, China; 4 Department of Neurology, Suzhou Industrial Park Xinghai Hospital, Suzhou, China

**Keywords:** Acute ischemic stroke, N-terminal pro-B-type natriuretic, Outcomes, CTP perfusion, Treatment decisions

## Abstract

**Objective:**

To investigate the impact of N-terminal pro-B-type natriuretic peptide (NT-proBNP) on CTP infarct core volume and poor 90-day functional outcomes in acute ischemic stroke (AIS).

**Methods:**

A total of 403 hospitalized patients with AIS in the Stroke Center of the First Hospital Affiliated to Soochow University were enrolled from March 2018 to January 2021. The association between NT-proBNP and clinical outcomes in acute ischemic patients was assessed by logistic regression and adjusted for confounding factors. Also, subgroup analyses were conducted based on treatment decisions.

**Results:**

NT-proBNP was positively correlated with CTP ischemic volume (*p* < 0.001), infarct core volume (*p* < 0.001), and ischemic penumbra volume (*p* < 0.001). Univariate analysis showed that the influence of NT-proBNP and functional outcomes were statistically significant in model 1 (*p* = 0.002). This phenomenon was persistent after adjusted for age, sex, and body mass index in model 2 (*p* = 0.011), adjusted for SBP, current smoking, family history of stroke, hypertension, and diabetes mellitus in model 3 (*p* < 0.001), and adjusted for TnI, D-dimer, PLT, Cr, TC, TG, HDL-C, treatment decisions, and NIHSS score in model 4 (*p* = 0.027). A high NT-proBNP was associated with a high 90-days mRS score among the total population, IV rt-PA, and standardized treatment groups, but not in IV rt-PA + EVT, EVT, and EVT/IV rt-PA + EVT groups.

**Conclusion:**

Elevated NT-proBNP levels reveal large CTP infarct core volume and poor 90-day functional outcome in AIS. NT-pro BNP is an independent risk factor for functional outcomes.

## Introduction

Stroke is the second leading cause of death and a major cause of disability worldwide with increasing incidence because of the aging population [1]. Interestingly, the elevated N-terminal pro-B-type natriuretic peptide (NT-proBNP) level in acute cerebral infarction is associated with heart failure (HF) [2]. Also, a strong interconnection between the heart and brain is recently emerging as a new discipline, neurocardiology [3, 4]. The heart and brain are not only the target but also endocrine organs, as components of a complex neuroendocrine reticular system [5]. This might explain the elevated BNP after stroke [6]. BNP is a vital marker of the activation of the human NP system and is expressed in the brain and the heart. Although the effect of BNP on cardiac insufficiency is widely accepted, the effect on the brain is underestimated. Few previous studies have explored the relationship between NT-proBNP levels and CTP ischemic volume and the underlying mechanisms explaining the effects of NT-proBNP levels on 90-day work in acute ischemic stroke (AIS).

The prognostic impact of CTP is currently inconclusive. Some studies demonstrated that CTP infarct core volume is an independent prognostic factor on functional outcome [7]. Some studies found that neither the volume of the penumbra nor ischemic core measured on CTP was associated with early neurological improvement [8]. Thus, it could be deduced that the time window for treatment options relies on linear growth of infarction, and the CTP ischemic volume plays a decisive role in the CTP ischemic penumbra volume. However, the infarct growth was not linear from symptom onset to baseline imaging in most patients [9]. This nonlinearity could be attributed to several influencing factors, such as the establishment of collateral circulation [10] and blood pressure [11]. Few previous studies have observed the correlations between NT-proBNP levels and the CTP ischemic volume, CTP infarct core volume, and CTP ischemic penumbra volume.

Intravenous thrombolysis and endovascular thrombectomy (EVT) are the standardized treatments for AIS worldwide [12]. A series of randomized controlled trials of EVT based on intravenous thrombolysis has yielded significant positive results for anterior circulation stroke within 6 h of onset, irrespective of patient characteristics [13]. During the prolonged window of 6–24 h, the results improved in patients with salvageable brain tissue, according to perfusion imaging [14]. Nonetheless, EVT is only indicated for a small subset of stroke patients, and a successful recanalization of EVT patients might not provide good functional outcomes [15]. However, few studies have focused on the effect of NT-proBNP on the outcomes after cerebrovascular revascularization treatment.

Therefore, this study aimed to investigate whether NT-proBNP is related to CTP ischemic volume correlation, whether to predict poor prognosis in AIS patients with different treatments, and to explore the underlying mechanisms by which NT-proBNP affects stroke outcomes.

## Methods

### Study design and population

**Fig. 1:**
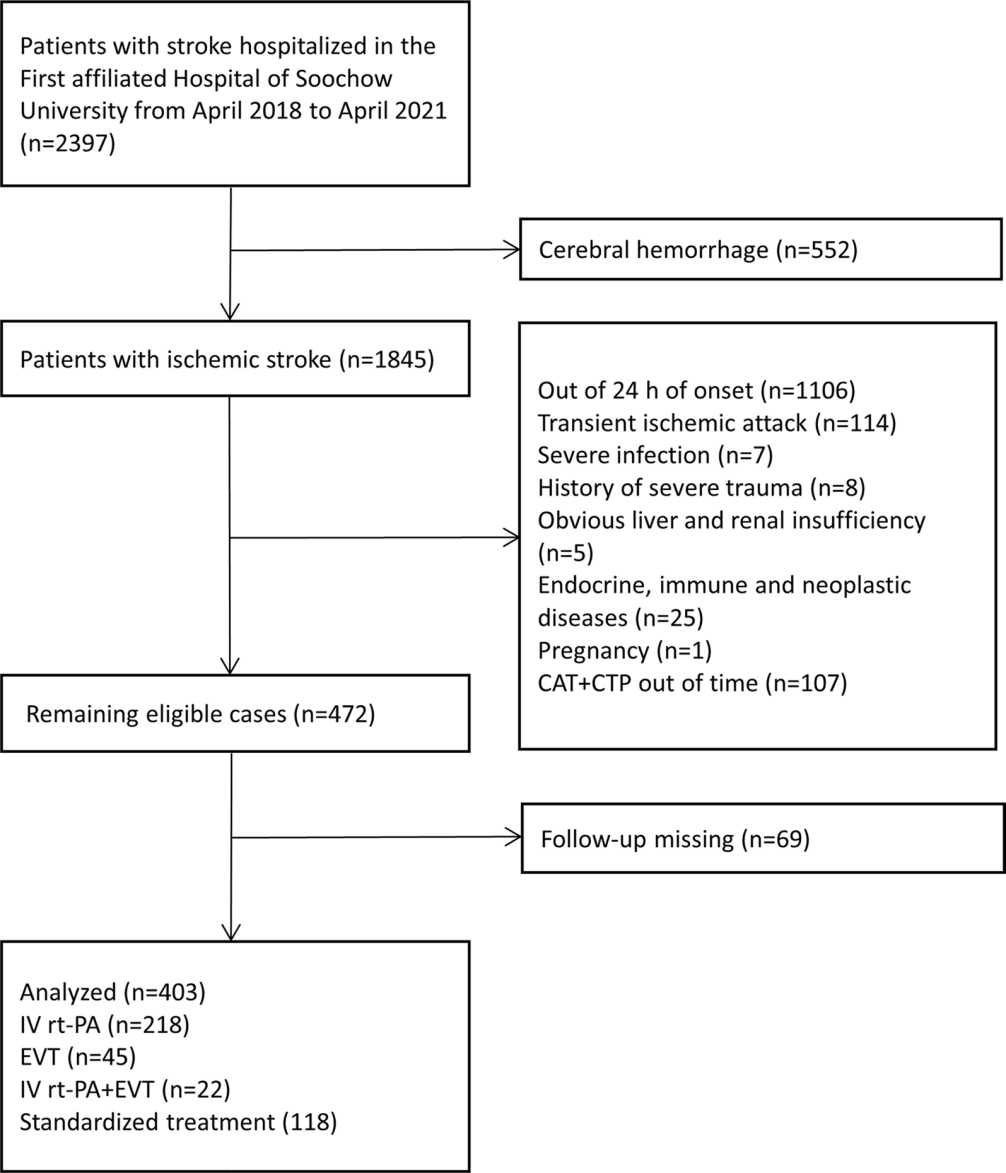
Flow diagram of included and excluded patients

The present study was conducted in the Stroke Center of the First Hospital Affiliated with Soochow University, followed by a retrospective analysis of the data collected prospectively from March 2018 to January 2021. Finally, 403 patients included in this study fulfilled all the following criteria: (1) Age ≥ 18-years-old; (2) The presence of acute ischemic lesions in the anterior circulation within 24 h of onset was confirmed by the imaging methods (magnetic resonance angiography (MRA) or computed tomography (CT); (3) Patients with acute onset for the first time or with previous cerebral infarction without obvious sequelae; (4) estimation of the NT-proBNP level and undergoing CTA + CTP within 0.5 h after admission to the hospital; (5) Ethics approval and consent to participate. The exclusion criteria were as follows: (1) Patients with cerebral hemorrhage or intracranial mass (cerebral hemorrhage, such as post-infarction hemorrhage after hospitalization, excluded by emergency cranial CT); (2) Patients with transient ischemic attack; (3) Patients with severe infection or septic shock; (4) Patients with a history of severe trauma and received surgical treatment; (5) Obvious liver and renal insufficiency; (6) Endocrine, immune, and neoplastic diseases; (7) Pregnancy (Fig. 1). All patients provided informed consent, and the data were analyzed anonymously. The ethical approval for this study was obtained from the ethics committees of the First Hospital Affiliated to Soochow University (No. 2019057).

### Data collection and definition

**Fig. 2:**
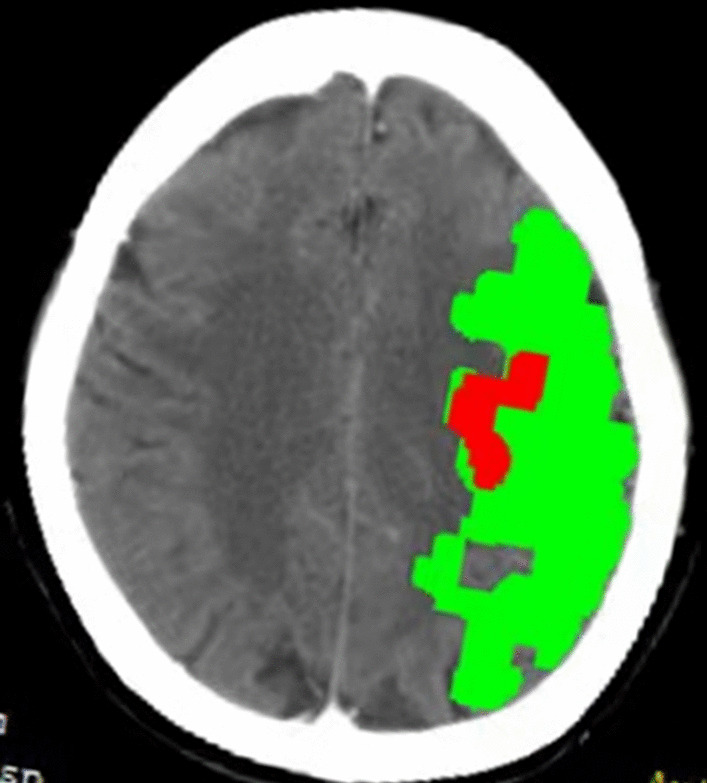
CT perfusion imaging images reconstructed by post-processing software MISta. CTP infarct core volume (Red): CBF＜30%. CTP ischemic penumbra volume (Green):Mismatch. CTP ischemic volume (Red plus Green): DT>3s^+^

Data, including the age, gender, individual addiction of smoking, previous history of hypertension, diabetes mellitus, stroke, family history of stroke, atrial fibrillation (AF) (previous AF and new-onset AF at stroke, AF is indicated by electrocardiography or continuous ECG monitoring when the patient arrives at the emergency department), and the clinical parameters obtained from the physical examination (height, weight, body mass index (BMI), systolic blood pressure (SBP), diastolic blood pressure (DBP), initial glucose, and the National Institute of Health Stroke Scale (NIHSS) score), were collected from medical records. Hypertension was defined as SBP ≥ 140 mmHg or DBP ≥ 90 mmHg. Diabetes mellitus was defined as fasting glucose ≥ 7.0 mmol/L, and the present treatment was insulin or an antidiabetic drug. Body mass index (BMI) was defined as weight in kilograms divided by the square of height in meters. The laboratory tests included serum triglycerides (TGs), total cholesterol (TC), low-density lipoprotein cholesterol (LDL-C), high-density lipoprotein cholesterol (HDL-C), and NT-proBNP. TOAST classification was divided into large-artery atherosclerosis (LAA), cardioembolic (CE) and other. LAA was defined as > 50% stenosis of the vessel lumen in extracranial or intracranial segment of internal carotid artery (ICA), M1/M2 segment of middle cerebral artery (MCA) or anterior cerebral artery (ACA).The functional outcome was assessed by a trained operator via face-to-face interview using the 90-day modified Rankin Scale (mRS) after the onset of symptoms. An excellent outcome was defined as the 90-day mRS score of 0–2, and a poor outcome was defined as a score of 3–6. The CTP ischemic penumbra volume reflected the collateral status. All perfusion images were post-processed on the commercial software MIStar (Apollo Medical Imaging Technology, Melbourne, Australia) using a single value deconvolution with delay and dispersion correction. The previously validated thresholds were applied to measure the CTP ischemic volume (delay time [DT] > 3 s) and CTP infarct core volume (relative cerebral blood flow (rCBF) < 30%). The CTP ischemic penumbra volume was calculated as follows: acute hypoperfused lesion volume minus the infarct core volume (Fig. 2).

### Treatment

Patients suspected to have had a stroke onset (or last seen in good condition) within 24-h window were subjected to multimode CT (NCCT + CT angiography + CT perfusion) examination by the neurologists. The patient’s treatment decisions (including intravenous thrombolysis both standard-dose thrombolysis (0.9 mg/kg) and low-dose thrombolysis (0.6 mg/kg), thrombectomy, thrombolysis and thrombectomy, no recanalization treatment) were made by experienced and senior neurologists.

### Outcomes

The functional outcome was assessed by mRS at 90 days after stroke (mRS 0–2 points as good prognosis and ≥ 3 points as poor prognosis), as determined by a trained operator blind to patient information using a validated telephone script.

### Statistical analysis

Kolmogorov–Smirnov test was used to assess the normality of numerical variables, while the median and interquartile range (IQR) were used to describe continuous variables in the non-normally distributed variables. The normative continuous variables were analyzed by independent sample’s t-test. The data were expressed as mean ± standard deviation (SD). The non-normative data were analyzed by Mann–Whitney U test. The categorical variable data were expressed as the count and percentage, and chi-square test or Fisher’s exact test was used for comparison between groups. Spearman’s rank correlation assessed the correlation between NT-proBNP and CTP perfusion status in the total population. The associations between the functional outcome variables and covariates were explored using univariate analysis testing each predictor and multivariable logistic regression analysis to adjust for potential confounders. For multivariate logistic regression analysis, we adjusted the confounding variables, including age, sex, BMI, baseline SBP, smoking, family history of stroke, hypertension, diabetes mellitus, TnI, D-dimer, PLT, Cr, TC, TG, HDL-C, treatment decisions, and baseline NIHSS score with a bivariate *p* < 0.10. A two-tailed *p*-value < 0.05 was considered significant. Also, subgroup analyses based on the classification of treatment decisions were conducted. NT-proBNP was grouped according to different cutoff values. Next, a receiver operating characteristic (ROC) curve was drawn to analyze the diagnostic value of NT-proBNP in the functional outcomes in different subgroups. All the data were analyzed using the SPSS software (IBM SPSS Statistics for Windows, version 26.0; IBM Corp, Armonk, NY, USA) and GraphPad Software (GraphPad Prism for Windows, version 9.0.0; San Diego, CA, USA). A two-tailed *p*-value < 0.05 indicated statistical significance.

## Results

### Baseline characteristics

Table 1 presents the clinical characteristics of the participants. Compared to the excellent functional outcome (mRS 0–2) group, a large number of patients in the poor functional outcome (mRS 3–6) group were older (*p* = 0.004), had higher TnI (*p* = 0.010), higher D-dimer, poor initial NIHSS score, greater CTP ischemic volume, greater CTP infarct core volume, greater CTP ischemic penumbra volume, and higher NT-proBNP (all *p* < 0.001). No difference was detected in the sex, BMI, baseline SBP, DBP, other biochemical variables, medical history, treatment decisions, and TOAST between the two groups (all *p* > 0.05).

**Table 1:** Baseline characteristics of the studied patient population and the single factor analysis as stratified by the prognosis of stroke (n = 403)

Variables	Total population (n = 403)	Functional outcome		x^2^/t/Z	*p*-value
		Excellent (90-day mRS 0–2) (n = 228)	Poor (90-day mRS 3–6) (n = 175)		
Age, median (IQR)—years	67 (56–75)	66.5 (55–73)	69 (60,77)	− 2.851	**0.004**
Sex—no. (%)				2.907	0.088
Male	256 (63.5)	75 (32.9)	72 (41.1)		
Female	147 (36.5)	153 (67.1)	103 (58.9)		
BMI, mean (SD)—kg/m^2^	24.48 ± 3.36	24.43 ± 3.57	24.43 ± 3.58	0.254	0.799
SBP, median (IQR)—mmHg	155 (138–175)	154 (137–173)	156 (141–178)	− 0.971	0.332
DBP, median (IQR)—mmHg	88 (78–100)	88 (77.25–99)	89 (78–101)	− 0.538	0.590
*Medical history*					
Atrial Fibrillation—no. (%)	121 (30.0)	52 (22.8)	69 (39.4)	13.019	** < 0.001**
Hypertension—no. (%)	269 (66.7)	144 (63.2)	125 (71.4)	3.051	0.081
Diabetes mellitus—no. (%)	99 (24.6)	50 (21.9)	49 (28.0)	1.969	0.161
Family history of stroke—no. (%)	86 (21.3)	51 (22.4)	35 (20.0)	0.331	0.565
Smoking—no. (%)	137 (34.0)	80 (35.1)	57 (32.6)	0.279	0.597
*Biochemical variables*					
TnI, median (IQR)—pg/ml	11.2 (7.42–17.23)	10.765 (7.32–14.64)	12.76 (7.69–21.46)	− 2.577	**0.010**
D-dimer, median (IQR)—ng/mL	0.36 (0.22–0.81)	0.31 (0.22–0.52)	0.53 (0.26–1.25)	− 5.451	** < 0.001**
PLT, mean (SD)—10^9^/L	198.92 ± 58.55	201.45 ± 58.70	195.63 ± 58.35	0.988	0.324
Cr, median (IQR)—mmol/L	66.8 (57.7–79)	67.75 (58.03–79.9)	65.2 (56–78)	− 1.162	0.245
TG, median (IQR)—mmol/L	1.24 (0.91–1.6)	1.28 (0.9–1.74)	1.17 (0.93–1.5)	− 1.610	0.107
TC, median (IQR)—mmol/L	4.3 (3.67–5.04)	4.21 (3.63–5.04)	4.36 (3.7–5.04)	− 1.158	0.247
HDL-C, median (IQR)—mmol/L	1.01 (0.86–1.19)	0.99 (0.84–1.16)	1.04 (0.88–1.21)	− 1.654	0.098
LDL-C, median (IQR)—mmol/L	2.72 (2.11–3.33)	2.7 (2.06–3.33)	2.78 (2.22–3.35)	− 1.076	0.282
HCY, median (IQR)—mmol/L	6 (5.6–7.2)	6 (5.6–6.9)	6.1 (5.5–8)	− 1.004	0.315
NT-proBNP, median (IQR)—100 pg/mL	1.46 (0.5–7.38)	1.02 (0.49–4.56)	3.28 (0.51–10)	− 3.492	** < 0.001**
*CTP imaging date*					
CTP ischemic volume (IQR)—mL	34 (0–110)	15.5 (0–58.5)	88 (15–185)	− 7.299	** < 0.001**
CTP infarct core volume (IQR)—mL	2 (0–14)	1 (0–6)	10 (1–59)	− 7.961	** < 0.001**
CTP ischemic penumbra volume (IQR)—ml	30 (0–84)	13 (0–51)	63 (12–102)	− 6.268	** < 0.001**
NIHSS—no. (%)				81.372	** < 0.001**
Excellent (0–4)	148 (36.7)	127 (55.7)	21 (12.0)		
Poor (5–42)	255 (63.3)	101 (44.3)	154 (88.0)		
Treatment decisions—no. (%)				3.725	0.293
IV rt-PA	218 (54.1)	132 (57.9)	86 (49.1)		
EVT	45 (11.2)	21 (9.2)	24 (13.7)		
IV rt-PA + EVT	22 (5.5)	12 (5.3)	10 (5.7)		
Standardized treatment	118 (29.3)	63 (27.6)	55 (31.4)		
TOAST—no. (%)				28.356	** < 0.001**
LAA	232 (57.6)	128 (56.1)	104 (59.4)		
CE	107 (26.6)	46 (20.2)	61 (34.9)		
Other	64 (15.9)	54 (23.7)	10 (5.7)		

*p*-value < 0.05 are shown in bold

*SBP* systolic pressure, *DBP* diastolic pressure, *NIHSS* National Institutes of Health Stroke Scale, *BMI* body mass index *p*-value, intergroup difference; *LAA* large-artery atherosclerosis, *CE* cardioembolic

### NT-proBNP and CTP perfusion status

**Fig. 3:**
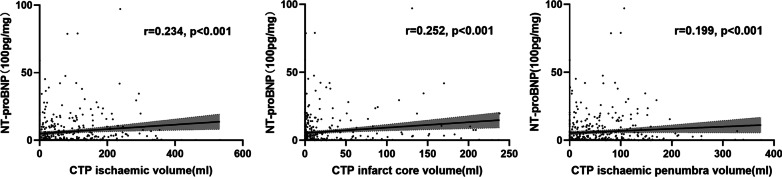
Three scatter plots for Spearman’s rank correlation analysis

Figure 3 presents Spearman’s rank correlation between NT-proBNP and CTP perfusion status in total population (n = 403). NT-proBNP was positively correlated with CTP ischemic volume (r = 0.234, *p* < 0.001), CTP infarct core volume (r = 0.252, *p* < 0.001), and CTP ischemic penumbra volume (r = 0.199, *p* < 0.001).

### NT-proBNP, CTP status, and functional outcomes

**Fig. 4:**
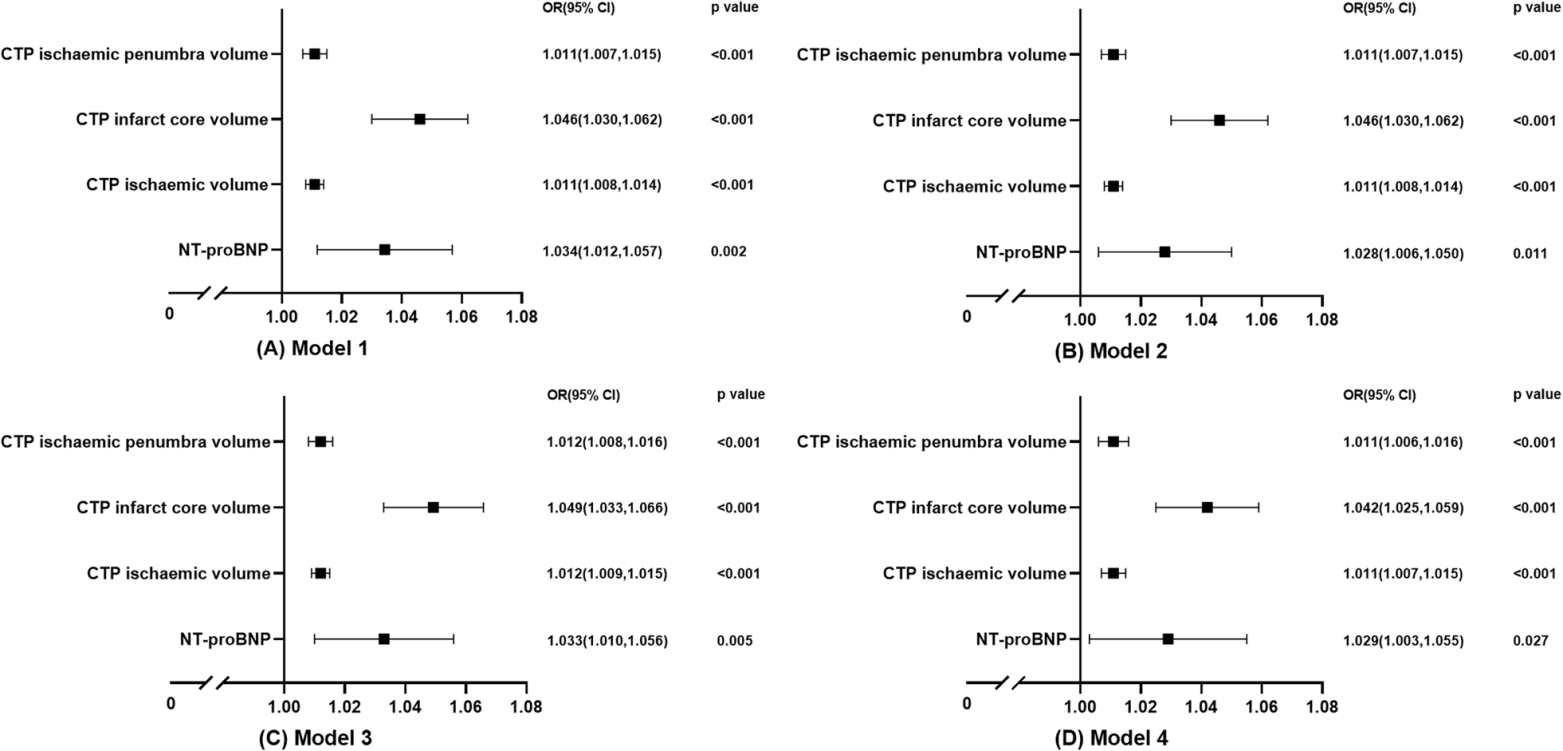
Bivariate logistics regression analysis of NT-proBNP on functional outcomes. Model 1: Unadjusted. Model 2: Adjusted for age, sex, and BMI. Model 3: Additionally adjusted for SBP, current smoking, family history of stroke, hypertension, and diabetes mellitus. Model 4: Additionally adjusted for TnI, D-dimer, PLT, Cr, TC, TG, HDL-C, treatment decisions, and NIHSS score

Subsequently, we performed logistic regression analyses on the factors associated with the 90-day mRS score (Fig. 4). The univariate analysis showed that the influence of NT-proBNP and functional outcome was statistically significant in model 1 (odds ratio (OR) = 1.034; 95% confidence interval (CI): 1.012–1.057; *p* = 0.002). The effect was persistent even after we adjusted for age, sex, and BMI in model 2 (adjusted OR = 1.028; 95% CI 1.006–1.050; *p* = 0.011), adjusted for SBP, current smoking, family history of stroke, hypertension, and diabetes mellitus in model 3 (adjusted OR = 1.033; 95% CI 1.010–1.015; *p* < 0.001), adjusted for TnI, D-dimer, PLT, Cr, TC, TG, HDL-C, treatment decisions, and NIHSS score in model 4 (adjusted OR = 1.029; 95% CI 1.003–1.055; *p* = 0.027). CTP ischemic volume, CTP infarct core volume, and CTP ischemic penumbra volume were associated with the functional outcomes in all models.

### NT-proBNP and functional outcomes

**Fig. 5:**
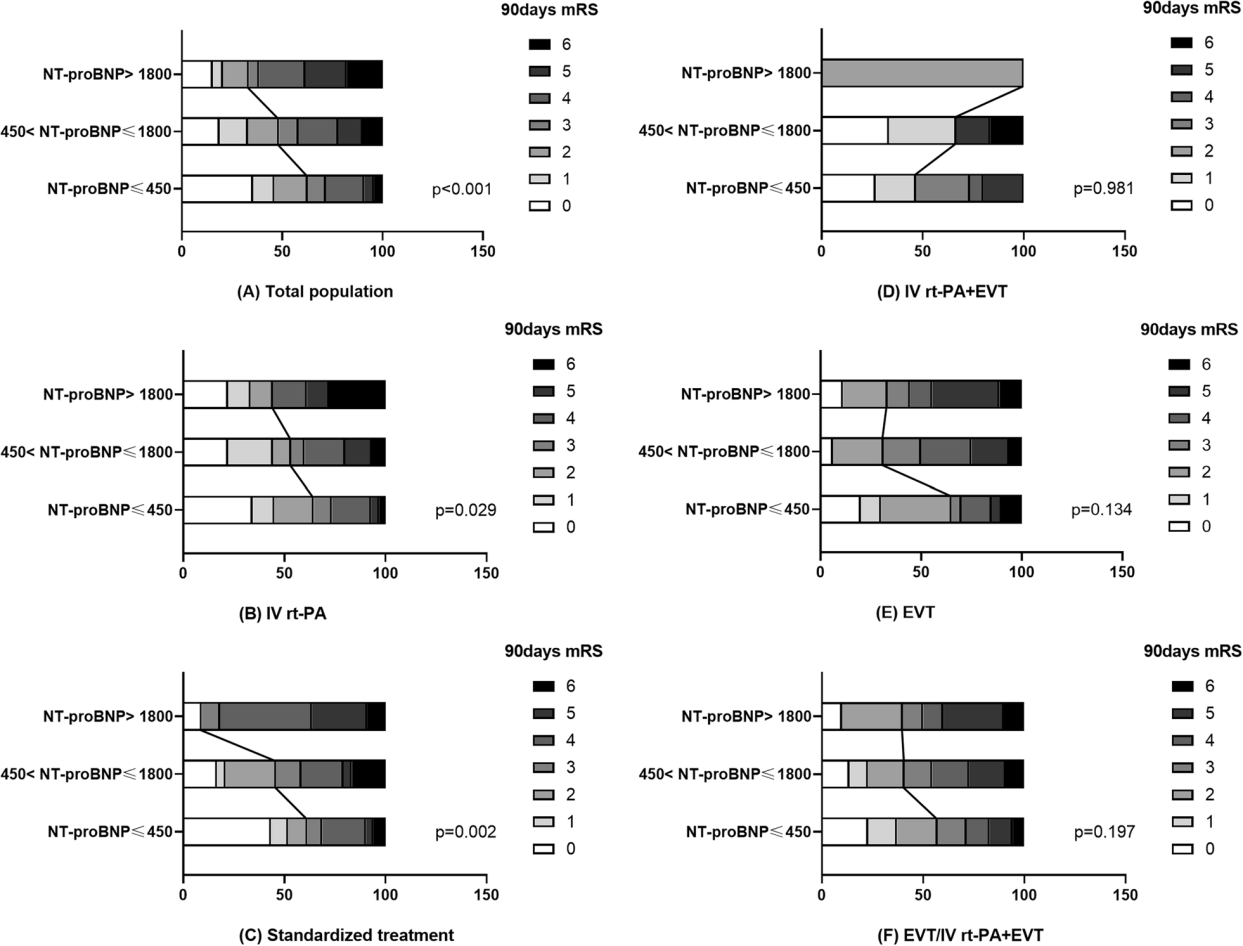
Three percentage stacked bar charts for the distribution differences of 90-day mRS

Figure 5 shows that the NT-proBNP group had higher 90-day mRS score among the total population group (*p* < 0.001, *H* = 25.01) (Fig. 5A), IV rt-PA group (*p* = 0.029, *H* = 7.09) (Fig. 5B), and standardized treatment group (*p* = 0.002, *H* = 12.93) (Fig. 5C). However, no significant differences were detected regarding excellent functional outcome among IV rt-PA + EVT (p = 0.981, *H* = 0.04) (Fig. 5D), EVT (*p* = 0.134, *H* = 4.03) (Fig. 5E), and EVT/IV rt-PA + EVT groups (*p* = 0.197, *H* = 3.25) (Fig. 5F).

### ROC of NT-proBNP on functional outcomes

**Fig. 6:**
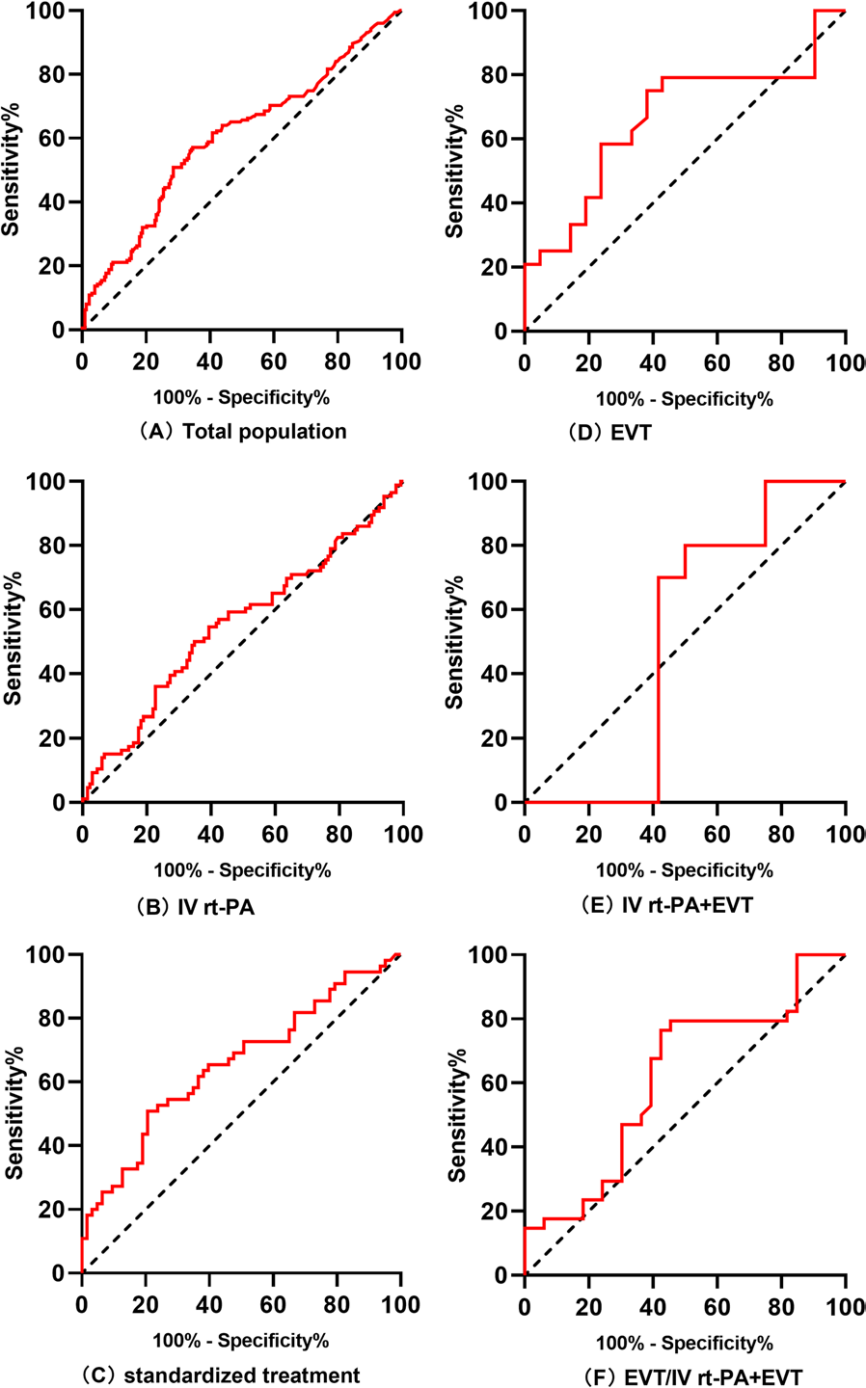
ROC of NT-proBNP on functional outcomes

Figure 6 presents the efficiency of the ROC curves in differentiating NT-proBNP in different treatment groups to predict the 90-day adverse outcomes. NT-proBNP had a predictive value in the total population group (area under the ROC curve (AUC) = 0.602, *p* < 0.001) (Fig. 6A) and standardized treatment group (AUC = 0.655, *p* = 0.004) (Fig. 6C) and no predictive value in the IV rt-PA group (AUC = 0.554, *p* = 0.181) (Fig. 6B), the IV rt-PA + EVT group (AUC = 0.508, *p* = 0.947) (Fig. 6D), the EVT group (AUC = 0.664, *p* = 0.061) (Fig. 6E), and the EVT/IV rt-PA + EVT group (AUC = 0.615, *p* = 0.104) (Fig. 6F).

## Discussion

BNP and NT-proBNP are the major components of the NP system and split into these forms for release from the atrial and ventricular myocardium following cardiomyocyte stimuli, such as volume overload, pressure overload, and ischemic injury [4, 16, 17]. BNP and NT-proBNP are commonly used in diagnosing or evaluating heart disease, and their role has been expanded and applied to stroke in recent years [18]. Reportedly, NT-proBNP is a biological marker of cerebrovascular disease for identifying ischemic stroke subtypes [19], predicting the incidence of atrial fibrillation-related stroke [20], and predicting stroke recurrence [21, 22]. The meta-analysis of prospective cohort studies suggested that NT-proBNP levels predict the prognosis for functional outcomes in ischemic stroke patients [23]. BNP is an active hormone with vasodilatory and diuretic effects and reduced left ventricular load [24]. Conversely, NT-proBNP is an inactive form with a longer half-life than BNP [25], and thus, a wide clinical application.

NT-proBNP levels are valuable predictors of stroke outcome [26]. A previous study showed that the levels of NT-proBNP were positively associated with the risk of ischemic and hemorrhagic stroke [2]. S prospective, multicenter observational study of 4215 patients with AIS pointed out that NT-proBNP is an independent prognostic marker of all-cause mortality in Chinese patients with AIS [27]. A study of 441 CE patients > 80-years-old with 90-day and 1-year follow-up demonstrated that BNP, but not s-cTnI, was an independent predictor of death [28]. Another study investigated 270 AIS patients aged 21–87 years, with symptoms within 48 h. High BNP levels were independently associated with high mortality and a poor 90-day prognosis [29], which is also evident in TIA. In a follow-up study of 929 patients, aged 64–83 years with TIA or minor stroke, with a median follow-up of 6.4 years, NT-proBNP was found to independently predict the all-cause mortality, with the maximal predictive value in the > 80-years-old age group [30]. However, the mechanism underlying this phenomenon is yet to be elucidated.

The current study demonstrated that elevated NT-proBNP levels are associated with poor 90-day functional outcomes in AIS. Thus, we proposed the following: (1) Elevated NT-proBNP has a specific effect on the brain. NT-proBNP reflects the cardiovascular burden [31] and might be the most reliable biomarker in HF with high specificity and sensitivity [32]. NT-proBNP is also a marker of AF burden. The heavier the AF burden, the higher the BNP level; this might predict the progression of AF: paroxysmal to persistent AF [33]. Although NT-proBNP does not appear in the CHA2DS2VASc scale for assessing the risk of AF-related cerebral embolism, a systematic review and meta-analysis on data from 2958 patients with ischemic stroke was retrieved from 16 studies showed that BNP could be an accurate diagnostic marker of CE [34]. CE is characterized by a large infarct size, severe neurological impairment, and poor prognosis [26, 35, 36]. It is a major subtype of ischemic stroke; thus, it was inferred that elevated NT-proBNP is consistent with the clinical features of CE. (2) Patients in the medical intensive care unit with cerebral hemorrhage [37], undergoing non-cardiac surgery [38], mechanical ventilation [39], and pulmonary embolism [40], have a poor prognosis with elevated NT-proBNP, which might also increase the non-specific risk of stroke patients [41]. (3) Risk stroke, similar to NT-proBNP increases, is increased with age [42–44]. Age is a predictor of mortality in Ischemic Stroke [45]. It is assumed that the older the age, the higher the NT-proBNP levels and the worse the stroke prognosis.

The volume of CTP predicted infarct core is associated with poor clinical outcome in AIS imaged within 8 h of onset [46]. The elevated CTP ischemic core volume is associated with poor outcomes and a lower likelihood of shift towards improved outcomes [47]. A large initial infarct volume is significantly associated with poor clinical outcomes in patients who underwent EVT because of early window stroke [48]. The current study found that elevated NT-proBNP levels are associated with large CTP infarct core volume, which is consistent with the finding that elevated NT-proBNP levels are associated with poor functional outcomes at 90 days.

Endovascular thrombectomy is one of the robust treatments for large vessel ischemic stroke [49]. Endovascular thrombectomy is crucial for reducing disability and improving the quality of life after large-vessel ischemic stroke [50, 51]. In this study, NT-pro BNP is an independent risk factor for functional outcomes, while endovascular treatment could counteract its role as a biomarker for predicting poor outcomes. This phenomenon indicated that cerebrovascular recanalization therapy, whether EVT, IV rt-PA + EVT, or IV rt-PA, can reverse the poor prognosis even if the NT-proBNP levels are elevated indicating poor prognosis. These findings reflected the positive impact of cerebrovascular revascularization therapy. NT-proBNP reflects cardiovascular burden and may decrease with a lower cardiovascular stress response after cerebrovascular revascularisation therapy for acute ischaemic stroke.This might explain the mechanism by which the results of subgroup analyses differ across treatment decisions.

Previous studies have observed the effects of NT-proBNP on all-cause mortality, short and long-term all-cause mortality, cardiac death, functional outcomes, and short and long-term functional outcomes of ischemic stroke patients [23]. There were also studies discussing the effect of NT-proBNP on the prognosis of different TOAST types of stroke, but no studies have been found to explore the predictive value of BNP on poor prognosis after different treatment decision interventions. The correlation between NT-proBNP and CTPischemic volume was also lack of research coverage. In this study, based on the decisive influence of CTP ischemic volume on stroke prognosis, the correlation between NT-proBNP and CTP ischemic volume was used to explain that elevated NT-proBNP levels are associated with 90-day functional outcomes in acute ischemic stroke.

## Limitations

Nevertheless, the present study has some limitations. This was a single-center retrospective study, which might have selection bias. Since the population in this study was of Asian descent, the results may not be applicable to other ethnic groups. Also, the secondary outcomes, including mortality and hemorrhage, need to be evaluated in future large-scale studies. Data on left ventricular ejection fraction and troponin levels were less complete in this study, so we did not include them in the statistical analysis and we will collect data in this area in a future prospective database build.

## Conclusions

Elevated NT-proBNP levels are associated with large CTP infarct core volume and poor 90-day functional outcome in AIS. NT-pro BNP is an independent risk factor for functional outcomes.

## Data Availability

The datasets generated and/or analyzed during the current study are not publicly available currently because the survey is part of an ongoing cohort, which has a limited access period due to the regulation of local technology bureau. However, the datasets will be available from the corresponding author on reasonable request.
